# MutS Homologue hMSH5: Recombinational DSB Repair and Non-Synonymous Polymorphic Variants

**DOI:** 10.1371/journal.pone.0073284

**Published:** 2013-09-04

**Authors:** Xiling Wu, Yang Xu, Katey Feng, Joshua D. Tompkins, Chengtao Her

**Affiliations:** School of Molecular Biosciences, College of Veterinary Medicine, Washington State University, Pullman, Washington, United States of America; University of Massachusetts Medical School, United States of America

## Abstract

Double-strand breaks (DSBs) constitute the most deleterious form of DNA lesions that can lead to genome alterations and cell death, and the vast majority of DSBs arise pathologically in response to DNA damaging agents such as ionizing radiation (IR) and chemotherapeutic agents. Recent studies have implicated a role for the human MutS homologue hMSH5 in homologous recombination (HR)-mediated DSB repair and the DNA damage response. In the present study, we show that hMSH5 promotes HR-based DSB repair, and this property resides in the carboxyl-terminal portion of the protein. Our results demonstrate that DSB-triggered hMSH5 chromatin association peaks at the proximal regions of the DSB and decreases gradually with increased distance from the break. Furthermore, the DSB-triggered hMSH5 chromatin association is preceded by and relies on the assembly of hMRE11 and hRad51 at the proximal regions of the DSB. Lastly, the potential effects of hMSH5 non-synonymous variants (L85F, Y202C, V206F, R351G, L377F, and P786S) on HR and cell survival in response to DSB-inducing anticancer agents have been analyzed. These experiments show that the expression of hMSH5 variants elicits different survival responses to anticancer drugs cisplatin, bleomycin, doxorubicin and camptothecin. However, the effects of hMSH5 variants on survival responses to DSB-inducing agents are not directly correlated to their effects exerted on HR-mediated DSB repair, suggesting that the roles of hMSH5 variants in the processes of DNA damage response and repair are multifaceted.

## Introduction

The MutS homologue hMSH5 is a member of the DNA mismatch repair (MMR) family of proteins [1]–[3]. Instead of functioning in the MMR pathway, accumulating evidence support the idea that hMSH5 homologues play an array of diverse functions ranging from meiotic recombination, maintenance of chromosome integrity, class switch recombination (CSR), to DNA double-strand break (DSB) repair and DNA damage signaling [4]–[16]. In addition, a recent study has indicated that hMSH5 also plays a role in mitochondria DNA repair [17]. Recombinant hMSH5 protein interacts with hMSH4–the only other MutS homologous protein possessing no apparent role in the process of MMR–to form a heterocomplex that can recognize several Holliday junction (HJ) intermediate structures resembling those arisen during recombinational DSB repair [13]. However, it is important to note that the expression patterns of hMSH5 and hMSH4 differ significantly in mitotic tissues – of which hMSH5 is broadly expressed in a variety of organs; in contrast, expression of hMSH4 is considerably limited [1]–[3], [5], [18], [19]. This observation suggests that hMSH5 may also act independently of hMSH4 through networking with other proteins. Indeed, it has been shown that hMSH5 interacts with hMRE11, hRad51, c-Abl, and the Holliday junction-recognizing protein HJURP [5], [20], [21]. Although the levels of protein expression in cells are often low (MOPED or the Model Organism Protein Expression Database), hMSH5 could undergo induction and become phosphorylated in cells treated with ionizing radiation (IR) or cisplatin [14], [15], [20], raising the possibility that this MutS homologue may play a role in the process of DSB repair.

Undoubtedly, accurate and timely repair of DSBs is essential for cell survival and development [22]. The necessity of prompt DSB repair is also reflected by the presence of multiple DSB repair mechanisms in mammalian cells, by which DSBs are properly sensed and repaired either by the rapid but error-prone non-homologous end joining (NHEJ) pathway or by the slow but accurate homologous recombination (HR) pathway [22]–[25]. In essence, whereas NHEJ is not restricted by cell cycle progression, HR-mediated DSB repair–mainly operates during S and G2 phases of the cell cycle–relies on the availability of homologous templates present on sister chromatids or homologous chromosomes [26]. The emerging molecular details and the increased numbers of new HR and NHEJ factors suggest that the DSB repair process is dynamically coordinated and controlled at multiple levels. A highly guarded DSB repair process is required not only for achieving appropriate DSB repair outcomes but also restricting aberrant HR or NHEJ activities [27]. It is conceivable that uncontrolled up-regulation of HR or NHEJ-mediated DSB repair can pose a major risk for genomic instability through ectopic recombination and chromosome translocation.

Recent studies support a role for hMSH5 in the repair of cisplatin-induced DSBs, in which hMSH5 deficiency has been shown to elevate cisplatin-induced G2 arrest and increase cisplatin-triggered γ-H2AX foci formation [15]. Evidence especially pertinent to the role of hMSH5 in HR is the observed hRad51-dependent cisplatin-induced hMSH5 foci formation [15]. In the current study, we have directly analyzed the role of hMSH5 in recombinational DSB repair by the use of an *in vivo* reporter system. Our data indicates that hMSH5 promotes recombinational DSB repair, in which DSB-triggered hMSH5 chromatin association is preceded by and relies on the assembly of hMRE11 and hRad51 at the proximal region to the DSB. Given the importance of appropriate DSB repair, subtle changes inherited to the polymorphisms of DNA repair genes can affect the capacity of DSB repair and thereby increasing the risk of cancer predisposition [28]. Hence, we have also analyzed the effects of a series of hMSH5 non-synonymous polymorphic variants on cell survival in response to DSB-inducing anticancer drugs.

## Materials and Methods

### Cell Culture and Transfection

The generation of the reporter cell line 293TLα/pMMR-IR3 was described previously [29]. Stable cell lines 293T/f-hMSH5^L85F^, 293T/f-hMSH5^Y202C^, 293T/f-hMSH5^V206F^, 293T/f-hMSH5^R351G^, 293T/f-hMSH5^L377F^ and 293T/f-hMSH5^P786S^ were generated by a similar procedure that has been previously described for 293T/f-hMSH5 [30]. All cell lines were maintained in DMEM/High Glucose (HyClone, Thermoscientific, Waltham, MA) supplemented with 5% FBS, 5% NBS, 100 units/ml penicillin and 100 µg/ml streptomycin.

### Cell Lysate Preparation

Harvested cells were rinsed with PBS before lysate preparation. Cells were lysed in CelLytic-M Mammalian Cell Lysis Reagent (Sigma-Aldrich, St. Louis, MO) supplemented with 10 mM NaF, 1 mM Na_3_VO_4_, and 1×protease inhibitor cocktail (Thermo Scientific). Soluble fractions were obtained after a 30 min centrifugation at 14,000 rpm.

### Co-Immunoprecipitation (Co-IP)

Stable cell lines harboring the wild-type hMSH5 and the series of hMSH5 variants were transiently transfected with pcDNA6/Myc-hMSH4 by the calcium phosphate approach. Cells were collected 40 hours after transfection for lysate preparation. Cell lysates of each preparation was incubated with 5 µl mouse α-Myc antibody (Cat. No. 631206, Clontech, Mountain View, CA) or mouse α-hMSH5 antiserum [14] at 4^o^C for 2 hours. Immunoprecipitates were captured by the use of 40 µl BSA-saturated 50% slurry of Protein A/rG Agarose beads.

### Western Blotting and Antibodies

Cell lysates or immunoprecipitated proteins were separated by 4–20% gradient SDS-PAGE, transferred onto nitrocellulose membranes, and immunoblotted with indicated antibodies. Mouse α-hMSH5 antibody [14] and goat α-hMSH4 antibody were generated by Zymed (Invitrogen, Grand Island, NY). α-c-Abl (554148) was purchased from BD Pharmingen (San Diego, CA). α-HA antibody (MMS-101P) was purchased from Covance (Princeton, New Jersey). α-α-tubulin antibody (T6199), α-actin (A2066), and α-Flag M2 antibody (F3165) were purchased from Sigma.

### ChIP Assay

Reporter cells were transiently transfected with the pCMV(I-*Sce*I)3xNLS construct [29], [31], [32] at 5 hours prior to ChIP analysis, which was performed with the EZ-ChIP kit (Millipore, Billerica, MA) according to manufacturer’s instruction. Antibodies used in the experiments included α-hMRE11 (NB100–142, Novus Biologicals Inc., Littleton, CO), α-c-Abl, α-hRad51 (14B4) (NB100–148, Novus Biologicals Inc), α-hMSH5 [30], and α-hMSH4 [19]. Rabbit IgG (Upstate) was used as a negative control, and α-acetyl histone H3 (Upstate) or α-RNAPII (Upstate) were used as positive controls. Immunoprecipitated DNA was purified and analyzed by quantitative PCR in triplicates.

### RNA Interference

The generation of pmH1P-based RNAi constructs was carried out as previously described [33]. The efficiency of RNAi-mediated gene silencing was validated by Western blotting, and those displaying greater than 50% knockdown were selected. The RNAi targets on various genes were hMRE11 sh-1 (5′-AGGGGTTCTTGGAGAAGAA) [33], hRad51 sh-1 (5′-AAGGAGAGTGC GGCGCTTC) [15], hMSH5 sh-2 [14], and hMSH4 sh-1 (5′-CAGGTGGTCTCTGCATCCA).

### Clonogenic Survival Assay

Stable cell lines 293T/f-hMSH5, 293T/f-hMSH5^L85F^, 293T/f-hMSH5^Y202C^, 293T/f-hMSH5^V206F^, 293T/f-hMSH5^R351G^, 293T/f-hMSH5^L377F^ and 293T/f-hMSH5^P786S^ were used to perform clonogenic survival analysis in triplicate 6-cm plates (500 cells were seeded in each plate). Sixteen hours after seeding, cells were treated with indicated doses of cisplatin, bleomycin, doxorubicin, or camptothecin for one hour. Treated cells were maintained in culture for approximately 10 days to allow colony formation, and colonies containing more than 50 cells were counted. Cisplatin, bleomycin, doxorubicin, and camptothecin were purchased from Sigma.

### HR Reporter Analysis of hMSH5 Variants

To generate a defined DSB, the plasmid pCBA-(I-*Sce*I) was transfected into the reporter cell line 293TLα/pMMR-IR3 by using an Amaxa Nucleofector (Lonza Group Ltd, Allendale, NJ), in which individual hMSH5 variant was co-expressed with I-*Sce*I. Approximately 75% of the transfected cells were harvested for Western blotting analysis at 24 hours post transfection. Recombination frequency (percent of RFP positive cells) was determined by FACS analysis of 100,000 cells at 6 days post transfection (FACSCalibur, Becton Dickinson).

### Ethics Statement

The Washington State University Institutional Review Board has approved the use of human cell lines in this study.

## Results

### hMSH5 Promotes HR-mediated DSB Repair

**Figure 1:**
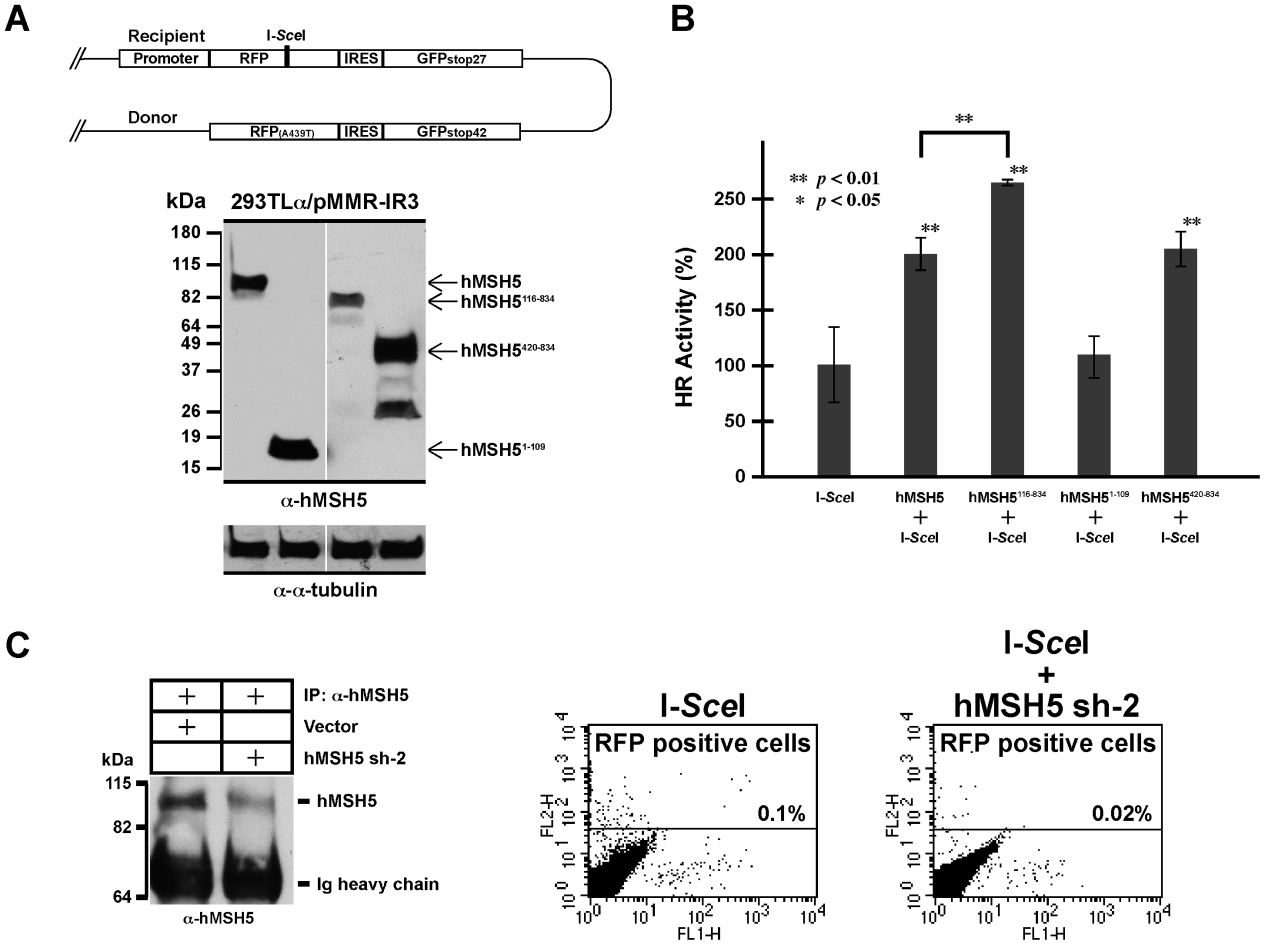
hMSH5 promotes recombinational DSB repair. (**A**) Schematically illustrated is the HR reporter locus in 293TLα (hMLH1 Tet-off) cells [29] by which the effects of hMSH5 on HR-mediated DSB repair were examined under the hMLH1 expression condition. Western blot analysis was performed to validate the expression of the full-length hMSH5 and three truncated hMSH5 fragments in 293TLα/pMMR-IR3 cells. (**B**) Effects of hMSH5 and truncation mutants on recombination. The full-length or truncated forms of hMSH5 were co-expressed with I-*Sce*I in 293TLα/pMMR-IR3 cells. Relative HR frequency (*i.e.* percentage of RFP positive cells) was determined by FACS analysis. Error bars represent standard deviations from the means of triplicate measurements. (**C**) RNAi-mediated hMSH5 knockdown compromises HR-mediated DSB repair. Reporter cells were treated with either I-*Sce*I or I-*Sce*I together with hMSH5 sh-2. The levels of hMSH5 knockdown by hMSH5 sh-2 were analyzed by Western blotting of immuno-concentrated hMSH5 from transfected 293TLα/pMMR-IR3 cells. The appearance of RFP positive cells (*i.e.* HR-mediated DSB repair) was determined by FACS analysis.

The role of hMSH5 in the process of recombinational DSB repair was first assessed by the HR reporter system 293TLα/pMMR-IR3 that measures the frequency of recombination initiated by an I-*Sce*I-induced DSB (Fig. 1A) [29]. 293TLα is an hMLH1 Tet-off cell line, in which the expression of hMLH1 can be repressed by doxycycline [34]. As described previously, the pMMR-IR3 locus contains two RFP and two GFP ORFs, of which one RFP ORF harbors the I-*Sce*I site with nucleotide deletions and the other RFP ORF has one nucleotide mutation, while both GFP ORFs possess internal stop codons [29]. Therefore, the production of RFP positive cells, following the induction of DSB by I-*Sce*I, is a direct consequence of recombinational DSB repair.

To test the role of hMSH5 in DSB-induced recombination, HR reporter analysis was first performed under the hMLH1 expression condition (*i.e.* in the absence of doxycycline). The results of the analysis indicate that hMSH5 transient expression increases the rate of recombination by at least 50% (Fig. 1B). To determine the region of hMSH5 that possesses the recombination promoting activity, three hMSH5 polypeptides were further tested (Fig. 1A). As shown in Fig. 1B, the carboxyl-terminal half of hMSH5 (aa420–834) exhibits a similar recombination promoting activity as that of the full-length protein, whereas the amino-terminal polypeptide (aa1–109) displays no obvious effect. Interestingly, hMSH5 fragment lacking the first 115 amino acids displays the strongest stimulatory effect on recombination (Fig. 1B), suggesting that the N-terminal portion of hMSH5 may negatively regulate its recombination-promoting activity. In addition, RNAi-mediated hMSH5 knockdown in the HR reporter cell line led to an approximately 5-fold reduction in recombination frequency (Fig. 1C). This effect of hMSH5 is hMLH1-independent as RNAi-mediated hMSH5 depletion in doxycycline-treated HR reporter cells could result in a similar level of reduction in recombination frequency (5.5-fold), although under this condition the overall recombination frequency was significantly increased (data not shown) [29]. Together, the results of these experiments suggest a direct role for hMSH5 in the process of DSB-induced recombination.

### The Formation of DSB Triggers hMSH5 Chromatin Recruitment

**Figure 2:**
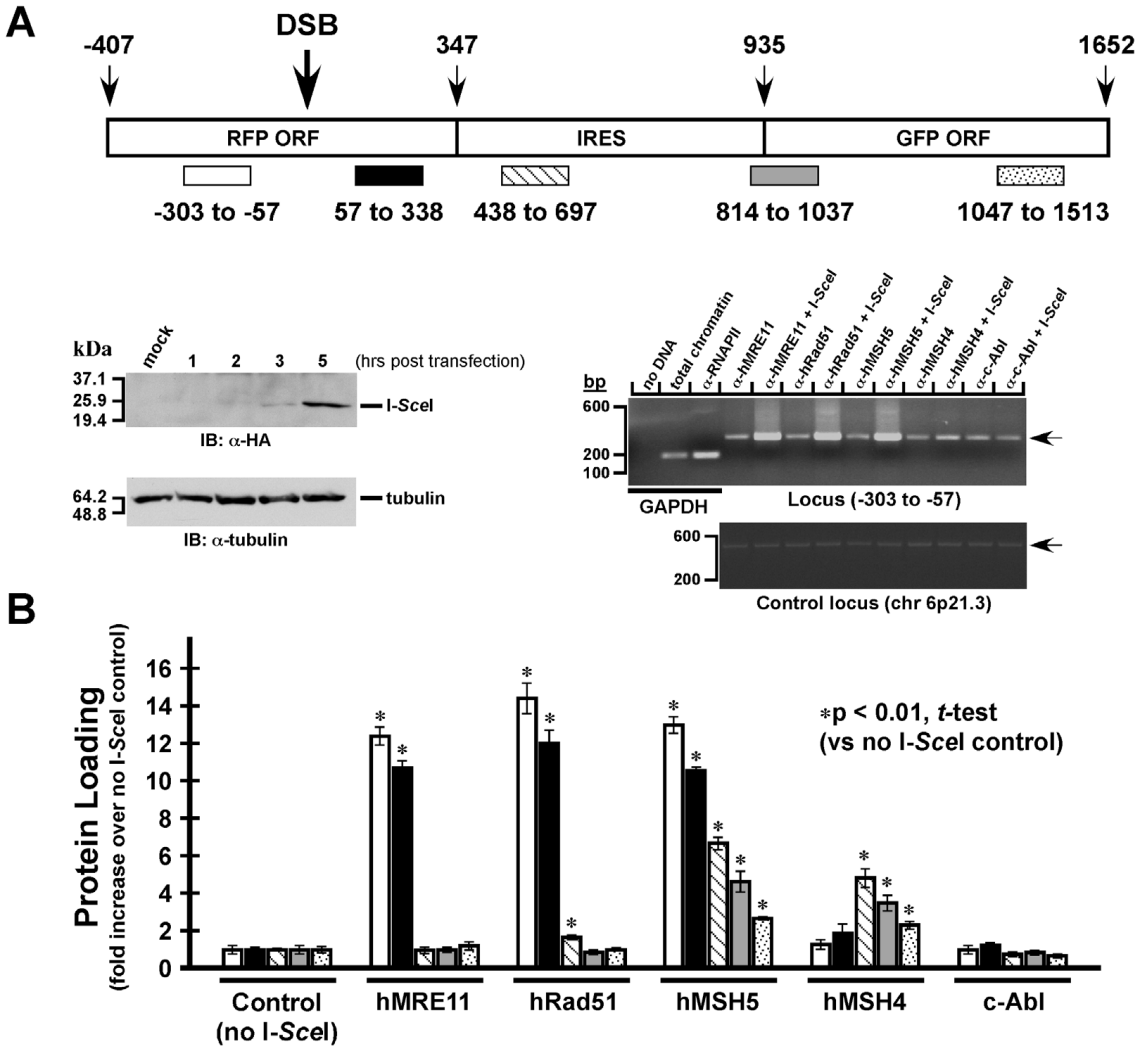
DSB triggers hMSH5 chromatin association. (**A**) DSB-triggered protein loadings at the proximal and distal regions. The regions, surrounding the site of I-*Sce*I, used for ChIP analysis were schematically illustrated. Numbers represent the distance from the site of I-*Sce*I in base pairs. The levels of I-*Sce*I expression, at different time points post-transfection, were analyzed by Western blotting with a α-HA antibody. Representative image of ChIP analysis of locus −303/−57 was shown, in which GAPDH was used as a positive control. PCR analysis (primer set: F13/IN2R1) of an unrelated region on 6p21.3 was included as an additional ChIP control. Arrows were used to mark the positions of the PCR products. (**B**) DSB-induced hMRE11, hRad51, hMSH5, hMSH4, and c-Abl loadings were analyzed at the proximal and distal loci. Error bars represent standard deviations from the means of triplicate measurements.

To directly analyze the role of hMSH5 in recombinational DSB repair, we next examined DSB-triggered loading of hMSH5 and several other DSB repair proteins at regions surrounding the site of I-*Sce*I-induced DSB using a chromosomally integrated reporter locus in 293T cells (Fig. 2A). This locus is identical to the aforementioned pMMR-IR3 locus except there are no mutations and internal stop codons in the second RFP ORF and the two GFP coding sequences, respectively. Thus recombination-provoked heteroduplex DNA would not contain mismatches, thereby eliminating interference from mismatch-triggered protein loading.

Specifically, protein loadings to five different regions surrounding the I-*Sce*I-induced DSB were investigated by chromatin immunoprecipitation (ChIP) (Fig. 2A). The DSB-triggered chromatin recruitment of hMSH5 and its binding partners hMSH4, hMRE11, hRad51, and c-Abl was monitored at 5 hours after I-*Sce*I transfection at which the expression of I-*Sce*I was readily detectable (Fig. 2A). In response to DSB formation, hMSH5, hMRE11, and hRad51 proteins were significantly enriched (>10-fold) at the proximal regions (*i.e.* −303/−57 or 57/338) (Fig. 2B). However, hMSH5 was also enriched in the distal regions where its levels of enrichment gradually decreased from 6.7-fold at region 438/697 to 2.7-fold at distal region 1047/1513 (Fig. 2B). This observation tends to suggest that the levels of hMSH5 loading may correlate with the decline of recombination frequencies over an increased distance from the break. Worthy of note is the similar protein loading patterns at the two proximal regions, suggesting bi-directional protein recruitment at the site of a DSB (Fig. 2B). DSB-induced hMRE11 and hRad51 loadings were only present at the proximal regions – consistent with their roles in the early stages of the recombination process [27], [35]. However, DSB-dependent hMSH4 chromatin enrichment only occurred at the intermediate and distal regions with the levels of protein recruitment decreasing gradually over the distance, ranging from 4.8-fold at region 438/697 to 2.3-fold at region 1047/1513 (Fig. 2B). In addition, ChIP analysis indicated that c-Abl was not enriched at any of the five regions examined (Fig. 2B). These data, collectively, support a dual role for hMSH5 in the process of recombinational DSB repair – an hMSH4-independent role at the proximal region and an hMSH4-dependent role at the intermediate and distal regions. Although the precise roles of hMSH5 in recombinational DSB repair are presently unclear, it is plausible that hMSH5 may function during the initial homology search as well as the processing of recombination intermediates.

### DSB-induced hMSH5 Chromatin Loading is hMRE11 and hRad51 Dependent

**Figure 3:**
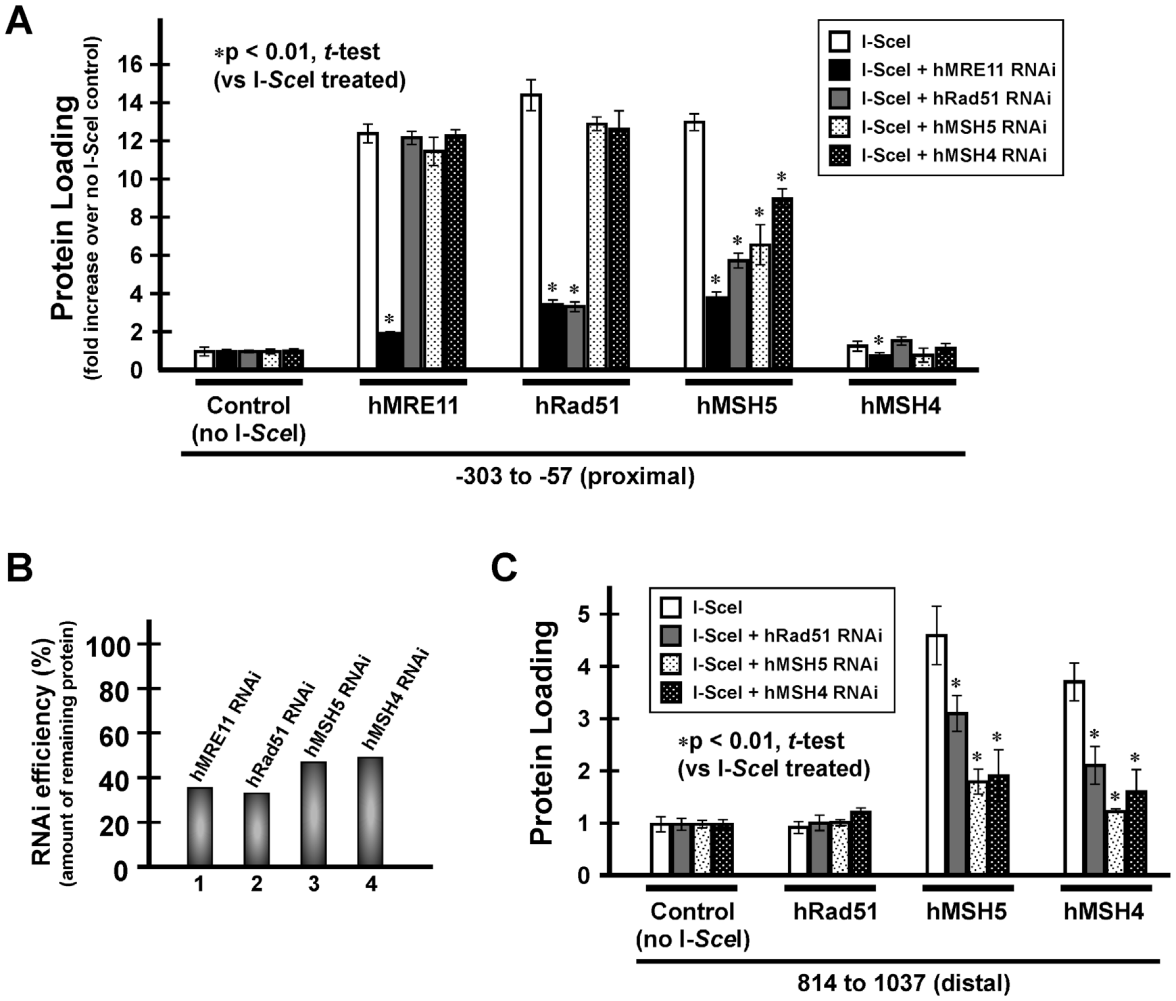
DSB-triggered hMSH5 loading on chromatin requires hMRE11 and hRad51. (**A**) ChIP analysis was performed in conjunction with RNAi-mediated gene silencing to determine the interdependency of DSB-triggered protein loadings at the proximal region. Controls without RNAi treatment were from Fig. 2B – the data is presented again on this graph for the purpose of comparison. (**B**) Knockdown efficiencies of shRNA encoding construct targeting hMRE11, hRad51, hMSH5, or hMSH4. Due to the difficulty in detecting endogenous hMSH4 in 293T cells by Western blotting, the hMSH4 knockdown efficiency was determined by the use of 293T/f45 cells. (**C**) ChIP analysis of the effects of RNAi on DSB-induced protein loadings at a distal region. Levels of protein loading in the absence of RNAi treatment were from Fig. 2B and included for the purpose of comparison. Error bars represent standard deviations from the means of triplicate measurements.

To determine the regulation of DSB-induced hMSH5 chromatin loading and to reveal the interrelationship among the hMSH5 interacting proteins, ChIP analysis was performed in conjunction with RNAi-mediated gene silencing at the proximal and distal loci (−303/−57 and 814/1037) (Fig. 3). The efficiencies of gene silencing by shRNAs were estimated to be at least 50% for all proteins examined (Fig. 3B). Consistent with the expected role of hMRE11 in the early phases of DSB repair [35], RNAi-mediated hMRE11 silencing significantly decreased DSB-induced hMRE11 and hRad51 protein loadings at the proximal region (Fig. 3A). In addition, silencing of hMRE11 was accompanied by the reduction of hMSH5 loading at both the proximal and distal regions as well as the loading of hMSH4 at the distal region (Fig. 3C). Conversely, DSB-triggered hMRE11 loading was not affected by RNAi-mediated silencing of hRad51, hMSH5, or hMSH4 genes (Fig. 3A), reiterating its upstream role in the process.

Likewise, RNAi-mediated hRad51 depletion led to the reduction of hMSH5 and hMSH4 loading similarly to that elicited by hMRE11 silencing; whereas the loading of hRad51 could only be affected by hMRE11 (Fig. 3). This observation is consistent with our previous finding that depletion of hRad51 by RNAi disrupts cisplatin-induced hMSH5 foci formation [15]. Furthermore, silencing of hMSH5 displayed no effect on hMRE11 or hRad51 loading at the proximal region, but it reduced DSB-triggered hMSH4 loading at the distal region (Fig. 3). Conversely, hMSH4 silencing led to a substantial reduction of hMSH5 loading at the distal region and a moderate decrease at the proximal region (Fig. 3). Together, these observations suggest that the actions of hMSH5 and hMSH4 in the process of DSB repair may not be completely overlapped, and hMSH5 appears to operate slightly upstream of hMSH4. Furthermore, the inhibitory effect of hMSH4 on NHEJ-mediated DSB repair can, at least indirectly, promote HR-based DSB repair [36].

### DSB-induced hMSH5 Chromatin Association is Abrogated by Y742F Mutation

**Figure 4:**
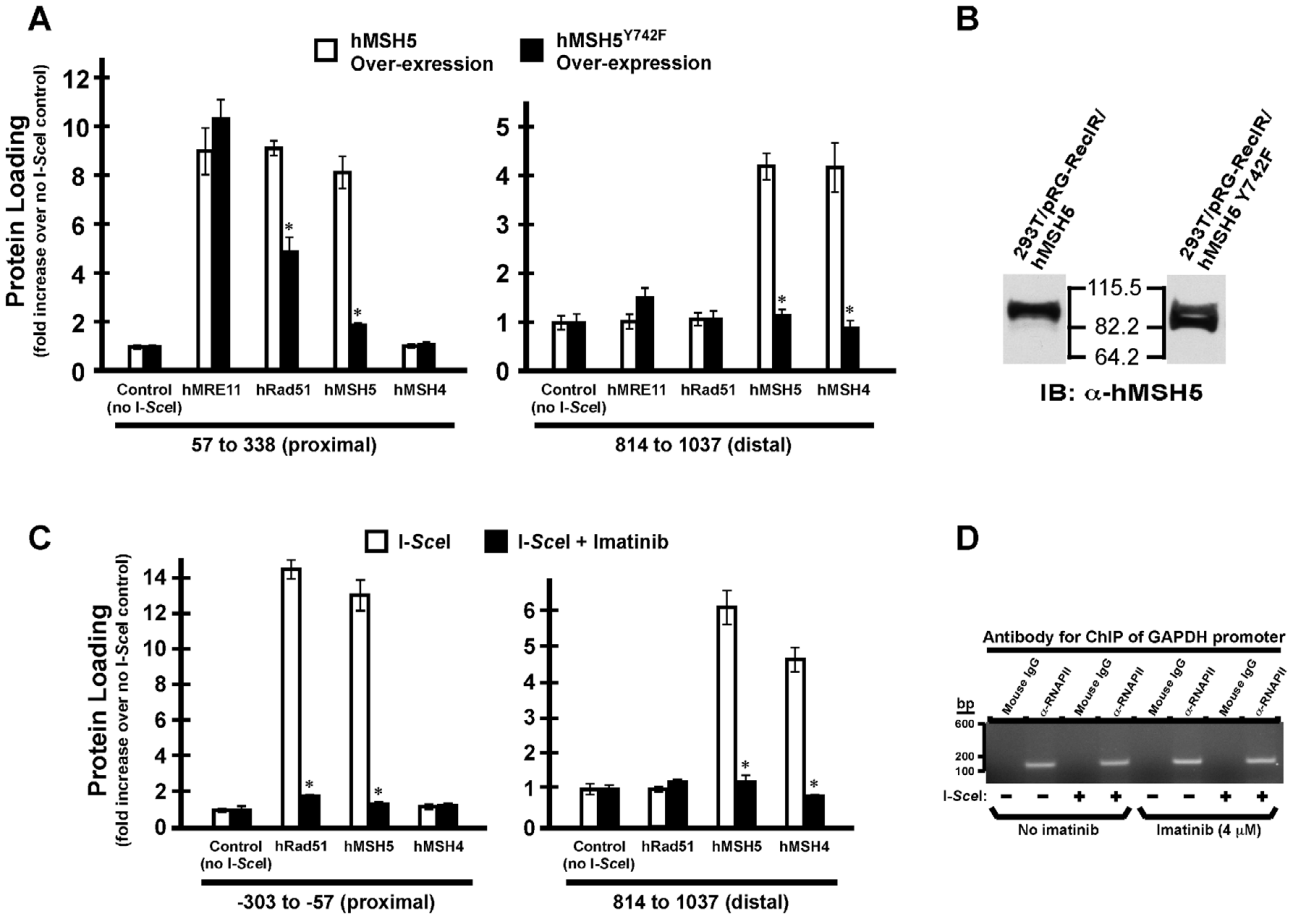
DSB-triggered hMSH5 chromatin association is disrupted by phosphorylation deficient mutant hMSH5^Y742F^. (**A**) ChIP analysis of the effects of hMSH5^Y742F^ on DSB-triggered protein loading at both the proximal and distal regions was carried out with 293T reporter cells expressing hMSH5 or hMSH5^Y742F^. Briefly, cells were transfected with pcDNA6/Flag-hMSH5 or Flag-hMSH5^Y742F^ and selected with 10 µg/ml blasticidin. (**B**) Expression of hMSH5 and hMSH5^Y742F^ in selected clones was validated by Western blot analysis of approximately equal numbers of hMSH5 and hMSH5^Y742F^ cells. (**C**) The effects of c-Abl kinase inhibition on DSB-induced protein loading at the proximal and distal regions. 293T reporter cells were pretreated with 4 µM imatinib for 48 hrs prior to the induction of DSB by I-*Sce*I transfection. ChIP analysis was performed to evaluate DSB-induced hRad51, hMSH5, and hMSH4 chromatin association. (**D**) ChIP analysis of GAPDH promoter performed with α-RNAPII or the mouse IgG in the presence or absence of imatinib treatment. Error bars represent standard deviations from the means of triplicate measurements. Asterisks indicate p<0.05 by Student’s *t*-test.

Disruption of hMSH5 tyrosine phosphorylation has been shown previously to impair cell survival and hMSH5 chromatin association in response to cisplatin treatment [15]. Thus, to assess the necessity of hMSH5 tyrosine phosphorylation in DSB processing, ChIP analysis was carried out to assess the effects of the phosphorylation deficient mutant hMSH5^Y742F^ (Fig. 4). We found that expression of the dominant negative hMSH5^Y742F^ resulted in a dramatic reduction of hMSH5 and hMSH4 loading at both the proximal and the distal regions (Fig. 4A), indicating that tyrosine742 phosphorylation was needed for DSB-induced hMSH5 assembly. Intriguingly, hRad51 loading, but not that of hMRE11, at the proximal region was also decreased to some extent by hMSH5^Y742F^ (Fig. 4A, left panel). Since hMSH5 acts downstream of hRad51 on DSB-containing chromatin (Fig. 3A) [15], the reduced hRad51 chromatin loading is unlikely mediated by hMSH5^Y742F^ directly. The disassembly of the hMSH5-c-Abl-hRad51 complex [5] following DSB induction could be impaired by the presence of hMSH5^Y742F^, thereby sequestering hRad51 from interacting with damaged chromatin. This is consistent with the observation that hMSH5 tyrosine phosphorylation disrupts the interaction between hMSH5 and c-Abl [14], [15], [20].

To further substantiate the need for c-Abl tyrosine kinase in the process of DSB repair, HR reporter cells were first treated with the c-Abl inhibitor imatinib and then were used to perform ChIP analysis of DSB-induced protein loading (Fig. 4C). Examination of a control GAPDH promoter locus following ChIP with α-RNAPII demonstrated that imatinib treatment had no effect on the loading of RNAPII (Fig. 4D). On the contrary, imatinib effectively blocked DSB-induced hRad51, hMSH5, and hMSH4 loading to corresponding proximal and distal regions (Fig. 4C). These results are consistent with the previous studies showing that c-Abl-mediated hRad51 phosphorylation on Tyr-315 is necessary for DNA damage-induced hRad51-chromatin association and subsequent DSB repair [37]–[39]. Together, we found that the c-Abl-mediated tyrosine phosphorylation event is essential for the association of hRad51 and hMSH5 with DSB-containing chromatin [15].

### Polymorphic hMSH5 variants Exert Distinct Effects on Cell Killing by Anticancer Agents

**Figure 5:**
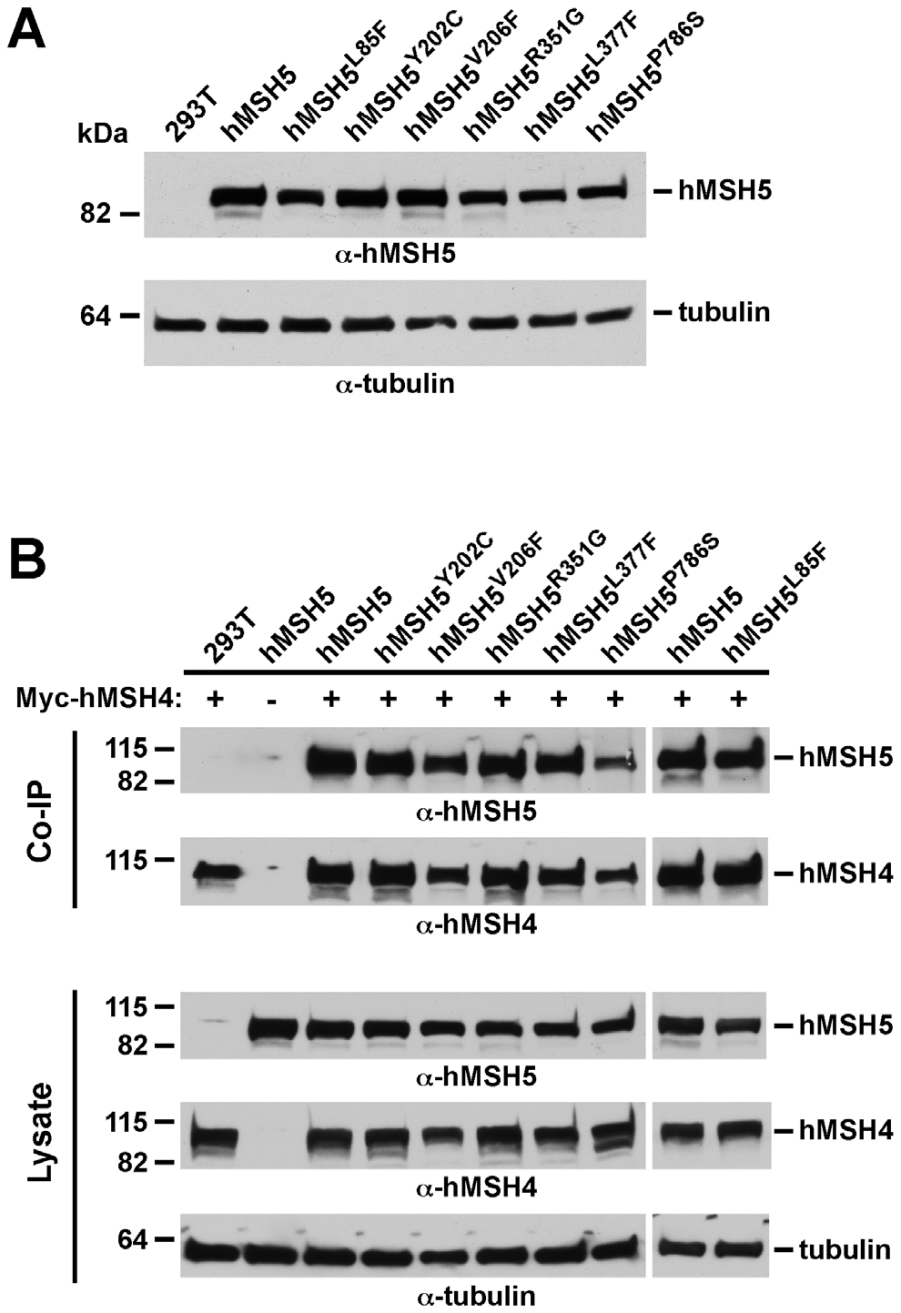
Polymorphic hMSH5 variants express at similar levels as the wild-type hMSH5 and interact normally with hMSH4. (**A**) Expression of the wild-type hMSH5 and six non-synonymous hMSH5 variants in 293T stable cell lines. Western blot analysis was performed with α-hMSH5 and α-tubulin antibodies. (**B**) Analysis of the effects of hMSH5 variants on the interaction of hMSH5 with hMSH4 by co-IP assay. Parental 293T and stable 293T clones expressing various forms of hMSH5 were transiently transfected to express Myc-hMSH4. Levels of protein expression in the cell lysates were examined by Western blotting performed with the mouse α-hMSH5, goal α-hMSH4, and α-tubulin antibodies. Co-IP was performed with the α-Myc antibody, and the levels of hMSH5 and hMSH4 in the immunoprecipitates were determined by immunoblotting with the mouse α-hMSH5 and goal α-hMSH4 antibodies.

The human hMSH5 gene is associated with several coding region non-synonymous polymorphisms [5], [40]; however, the functional significance of these polymorphic variants has not been investigated experimentally. This is an important issue because common genetic variations in DNA damage response and repair genes can affect DNA repair and cancer predisposition [41]. To this end, taking advantage of the fact that endogenous hMSH5 is normally expressed at a low level in 293T cells [14], we created a series of 293T stable cell lines expressing the wild-type and polymorphic variants of hMSH5. As shown in Fig. 5A, hMSH5 variants were all expressed at comparable levels in the established stable cell lines, although the expressions of hMSH5^L377F^ and hMSH5^P786S^ appeared to be slightly lower than the others. The physical interactions between hMSH4 and hMSH5 variants were then analyzed as a way to evaluate their functional changes. Specifically, cells were transiently transfected to express the hMSH5 interacting partner hMSH4, and the interaction was analyzed by co-IP. Clearly, after normalizing with the levels in the lysates, none of the hMSH5 variants significantly altered the interaction between hMSH5 and hMSH4 (Fig. 5B), suggesting that these hMSH5 non-synonymous alterations do not cause any substantial changes in the interaction domain.

**Figure 6:**
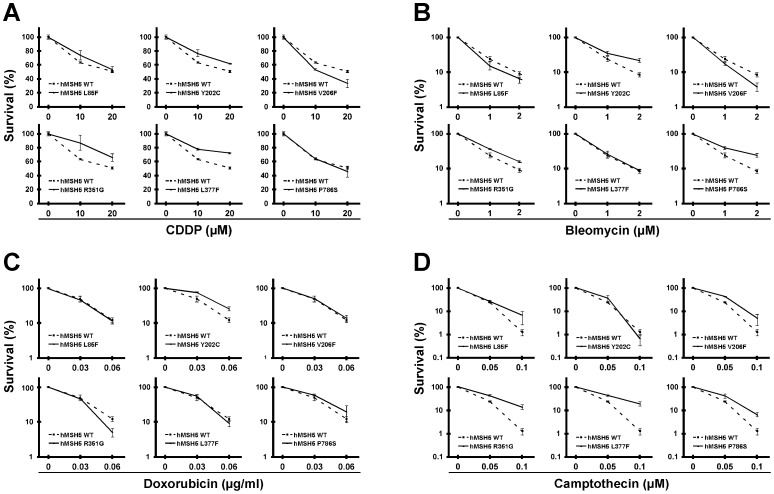
Polymorphic hMSH5 variants differentially affect cell survival in response to DSB-inducing anticancer agents. (**A**) CDDP. (**B**) bleomycin. (**C**) doxorubicin. (**D**) camptothecin. Clonogenic survival analysis of 293T stable cell lines, expressing individual hMSH5 polymorphic variants, was performed after cells were treated with indicated doses of DSB-inducing anticancer drugs. Cells expressing the wild-type hMSH5 were analyzed in parallel and were used as references. Error bars represent standard deviations from the means of triplicate measurements.

Next, clonogenic survival analysis was performed to assess the potential effects of hMSH5 variants on cellular responses to common anticancer agents. Survival responses of stable hMSH5 variant cell lines were analyzed after exposure to DSB-inducing agents cisplatin (CDDP), bleomycin, doxorubicin, and camptothecin. In comparison to that of the hMSH5 wild-type controls, hMSH5^Y202C^, hMSH5^R351G^ and hMSH5^L377F^ cells were resistant to CDDP, whereas hMSH5^V206F^ cells were sensitive to CDDP (Fig. 6A). In response to bleomycin treatment, cells containing hMSH5^Y202C^, hMSH5^R351G^ and hMSH5^P786S^ showed resistant phenotypes, while hMSH5^V206F^ cells were sensitive (Fig. 6B). Among cells treated with doxorubicin, only hMSH5^Y202C^ showed a significant resistant phenotype, while hMSH5^R351G^ displayed a sensitive response (Fig. 6C). Finally, in reference to that of the wild-type, hMSH5^V206F^, hMSH5^R351G^, hMSH5^L377F^ and hMSH5^P786S^ cells were all highly resistant to camptothecin (Fig. 6D). These observations indicate that the polymorphic hMSH5 variants can differentially modulate cellular responses to various DSB-inducing anticancer therapies – highlighting the potential impacts of hMSH5 variants on the effectiveness of chemotherapy.

### hMSH5 Polymorphic variants Affect Recombinational DSB Repair

**Figure 7:**
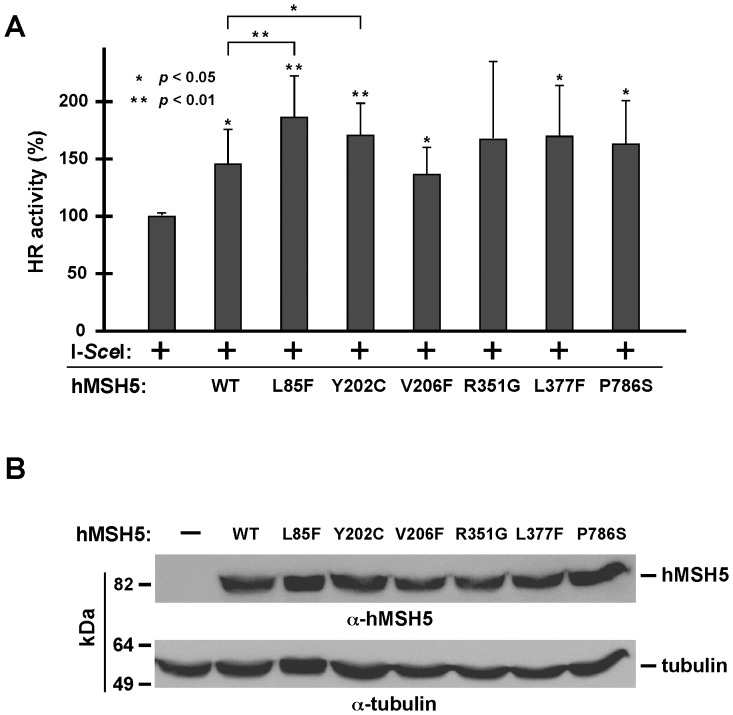
Polymorphic hMSH5 variants exert different effects on recombinational DSB repair. (**A**) 293TLα/pMMR-IR3 reporter cells were transfected to express I-*Sce*I and each of the polymorphic hMSH5 variants. The effects on HR were determined and quantified by FACS analysis of RFP positive cells. Error bars represent standard deviations from the means of five independent measurements. (**B**) Expression levels of the wild-type hMSH5 and polymorphic hMSH5 variants were determined by Western blot analysis. The levels of α-tubulin were also examined as loading controls.

To determine whether the altered survival responses mediated by hMSH5 variants associate with their effects on recombinational DSB repair, we analyzed the effects of these variants on DSB-induced recombination. The results of HR reporter analysis indicated that expression of the wild-type hMSH5 promoted recombinational DSB repair (Fig. 7A). To certain extents, all of the hMSH5 variants, except for hMSH5^R351G^, also promoted recombinational DSB repair (Fig. 7A). In particular, the hMSH5^L85F^ and hMSH5^Y202C^ variants could increase the recombination frequency above that of the wild-type hMSH5 (Fig. 7A). However, a clear correlation between the survival response and recombinational DSB repair has not been observed for this series of hMSH5 polymorphic variants. Collectively, these results infer that cancer cells possessing certain hMSH5 polymorphic variants will likely show altered cellular response to DSB-inducing anticancer agents; in which modulation of HR may not be the only underlying factor.

## Discussion

The ability to effectively repair DSBs is essential for cell survival, and defective DSB repair is a known factor for cancer development in humans [22]. Our current study has demonstrated an important role for hMSH5 in recombinational DSB repair. This role of hMSH5 is dependent on both hMRE11 and hRad51– likely mediated through protein interactions. Our findings support a scenario in which hMSH5 plays at least two separate roles in the process of recombinational DSB repair. The first role is c-Abl-dependent and it is immediately preceded by the action of hRad51 at the proximal region, where it, presumably, facilitates homology searching and heteroduplex formation. The second action for hMSH5 is at the intermediate and distal regions and it appears to be hMSH4-dependent, which ostensibly could be involved in harnessing the stability of recombination intermediates.

It is interesting to note that hRad51 is not at the distal region; as yet it controls hMSH5 loading at the distal region (Fig. 3C). This raises a possibility that hMSH5 may be loaded onto the damaged chromatin through the ends of DSBs and then outspread towards the distal regions. This view is consistent with the observed gradual decline of chromatin-associated hMSH5 while moving away from the site of DSB. Although tyrosine phosphorylation of hMSH5 is necessary for DSB-induced hMSH5 chromatin association [15] (Fig. 4A), this post-translational modification also disrupts the hMSH5-hMSH4 association [20]. Thus, conceivably, through a yet-to-be-identified mechanism, dephosphorylation of hMSH5 has to occur while translocating to distal regions to recruit hMSH4 (Fig. 3B). Alternatively, hMSH5 outspreading may be controlled by dephosphoryation, and the HJ-binding propensity of hMSH4-hMSH5 may facilitate their chromatin association at the distal regions. Based on the analysis of hMSH4-hMSH5 heterotypic and homotypic interactions [30], [42]–[44], it is very likely that hMSH4-hMSH5 may exist as a multimeric protein complex, such as a tetramer, for interacting with recombination intermediate structures through hMSH5.

Furthermore, our data suggest that hMSH5 acts slightly upstream of hMSH4. This is consistent with the mouse model postulating that Msh5 functions upstream of Msh4 in meiotic recombination [11]. It is worthy of note that hMSH4-hMSH5 interacts with GPS2 only in the heterodimeric state [42], whereby this interaction is especially relevant to recombination intermediates that may encompass several kilobases of homologous DNA [45]. In particular, the GPS2-HDAC3 complex may provide the necessary means for proper chromatin remodeling to facilitate the processing of recombination intermediates.

An intriguing observation was made during the course of these studies – in the absence of the homology donor sequence, a profound reduction of DSB-induced hRad51 and hMSH5 loading, but not hMRE11, was detected at the proximal region (Tompkins and Her, unpublished data). This observation tends to suggest that the loading of hRad51 and hMSH5 can also be regulated by the availability of the donor template. Development of a procedure allowing instantaneous DSB induction at a defined locus, for monitoring precise time-dependent loading patterns of various repair proteins, would be important to address how the existence of a homology donor influences the choice of DSB repair pathway.

Our examination of six non-synonymous hMSH5 variants has indicated that these polymorphic variants could exert distinct effects on cellular responses to different anticancer agents. Although DSB is commonly generated in cells treated with CDDP, bleomycin, doxorubicin, or camptothecin, the process of DSB induction in each case is mechanistically different and may also be cell cycle dependent. For instance, CDDP creates a variety of DNA lesions, of which the interstrand crosslinks are probably responsible for creating DSBs at replication forks [46]–[50]; likewise, the induction of DSB by camptothecin is S phase dependent [51]. However, it appears that doxorubicin acts mainly during the G2 phase of the cell cycle, whereas bleomycin generates DSBs in a relatively sequence specific manner [52]–[54]. Of note, these anticancer agents have very different chemical properties and create unique sets of DNA lesions that may activate multiple DNA repair and damage response pathways. These differences may attribute to the observed diverse cellular responses to anticancer agents mediated by different hMSH5 variants (Fig. 6). Among the six hMSH5 variants, hMSH5^Y202C^ is unique in several aspects. It stimulates recombination and renders cells resistant to CDDP, bleomycin and doxorubicin, and this variant is also predicated to be deleterious by the SIFT analysis (sift.bii.a-star.edu.sg; [55]). However, a clear correlation between the effects on recombination and cell survival cannot be made for the other hMSH5 variants, indicating that these variants may affect different aspects of the DNA damage response processes, *e.g.* NHEJ-mediated DSB repair and the induction of apoptosis. Obviously, further studies will be needed to decipher how hMSH5 variants impact cellular responses to anticancer therapy. Taken together, our current study has demonstrated a role for hMSH5 in recombinational DSB repair. The outcomes of this study should make it possible to explore the detailed molecular mechanisms involved with hMSH5 as well as the effects of various hMSH5 variants on cancer predisposition and cellular resistance to chemotherapeutics.
